# Allergic diseases of the skin and drug allergies – 2033. Metronidazole skin testing associated with systemic reaction

**DOI:** 10.1186/1939-4551-6-S1-P119

**Published:** 2013-04-23

**Authors:** Chwee Ying Tang, Teck Choon Tan, Hiok Hee Chng

**Affiliations:** 1 Rheumatology, Allergy and Immunology, Tan Tock Seng Hospital, Singapore

## Background

Type I hypersensitivity reaction to metronidazole and its skin testing are rarely reported. A 69 year old lady had laproscopic cholecystectomy on 27^th^September 2010. Post surgery, IV ciprofloxacin 400mg and metronidazole 500mg were infused at 8.00 pm. Within 30 minutes, she experienced pruritus and noted erythema on her left forearm followed by urticaria over upper limbs and chest. She reported mild shortness of breath and dizziness. There was no hypotension or angioedema. This was her first such reaction to drugs. Both antibiotics were stopped and IV hydrocortisone and oral chlorpheniramine prescribed. She was referred to our allergy service for suspected allergy to either ciprofloxacin or metronidazole. On assessment, she had received antibiotics on several occasions in the past and had reacted to ceftriaxone with pruritic maculopapular rashes. She had consumed metronidazole in June 2009 and ciprofloxacin in June 2009 and July 2010. A clinical diagnosis of Type I allergy to metronidazole was made.

## Methods

She underwent skin prick(SPT) followed by intrademal(ID) testing in February 2011.

## Results

At about 11.50 am, immediately after ID reading (test solution at 5mg/ml concentration), she complained of generalized pruritus including at the intradermal site. The ID reaction was an erythema of 8x8 cm compared to diluent which was 4x5cm at this highest concentration. The wheals were 3x4 mm for both. ID at 1:10 and 1:100 had not produced any erythema nor significant wheal. SPT was negative at up to 5mg/ml of metronidazole. Examination revealed erythema on forehead, urticaria (1x4cm) on left flank and a small urticaria on right flank. There was no dyspnoea. Chest auscultation and vital signs were normal. She was given IM phenergan 25 mg and all lesions resolved by 1.45 pm.

## Conclusions

We describe a case of metronidazole anaphylaxis with systemic reaction on skin testing although the ID reaction by criteria would have been considered negative. This case is a reminder that all proper precautions must be taken in skin testing.

